# Rare Case of OHVIRA with Ectopic Inguinal Hemiuterus and Ovary

**DOI:** 10.1055/a-2590-6030

**Published:** 2025-05-08

**Authors:** Stefanie Welke, Ferdinand Kosch, Andreas Müller, Amelie Hufnagel-Schmude, Leonie Rother, Verena Ellerkamp

**Affiliations:** 1 Department of Pediatric Surgery and Pediatric Urology, Municipal Hospital Karlsruhe, Karlsruhe, Germany; 2 Department of Obstetrics and Gynecology, Karlsruhe Municipal Hospital, Moltkestrasse, Karlsruhe, Germany; 3 Department of Radiology, Institute for Pediatric Radiology, Municipal Hospital Karlsruhe, Karlsruhe, Germany; 4 Department of Pediatric Surgery and Pediatric Urology, Eberhard Karls Universitat Tubingen, Tubingen, Germany

**Keywords:** OHVIRA syndrome, hemiuterus, hemivagina, vaginal obstruction

## Abstract

A 12-year-old girl presented with inguinal swelling and recurrent groin pain since menarche. Ultrasound showed an inguinally located ovary with normal perfusion. Herniorrhaphy revealed an ectopic inguinal left ovary with fallopian tube and atretic hemiuterus and a closed internal inguinal ring. Laparoscopy revealed a right-sided hemiuterus and vaginally palpable cervix, leading to the diagnosis of ectopic OHVIRA syndrome type 1.2. The left hemiuterus was resected and the left ovary was pulled through the inguinal canal into the abdomen. During 12 months of follow-up, the left ovary showed normal perfusion and sonomorphologic appearance, menstrual periods were uneventful.

## Background

**Fig. 1:**
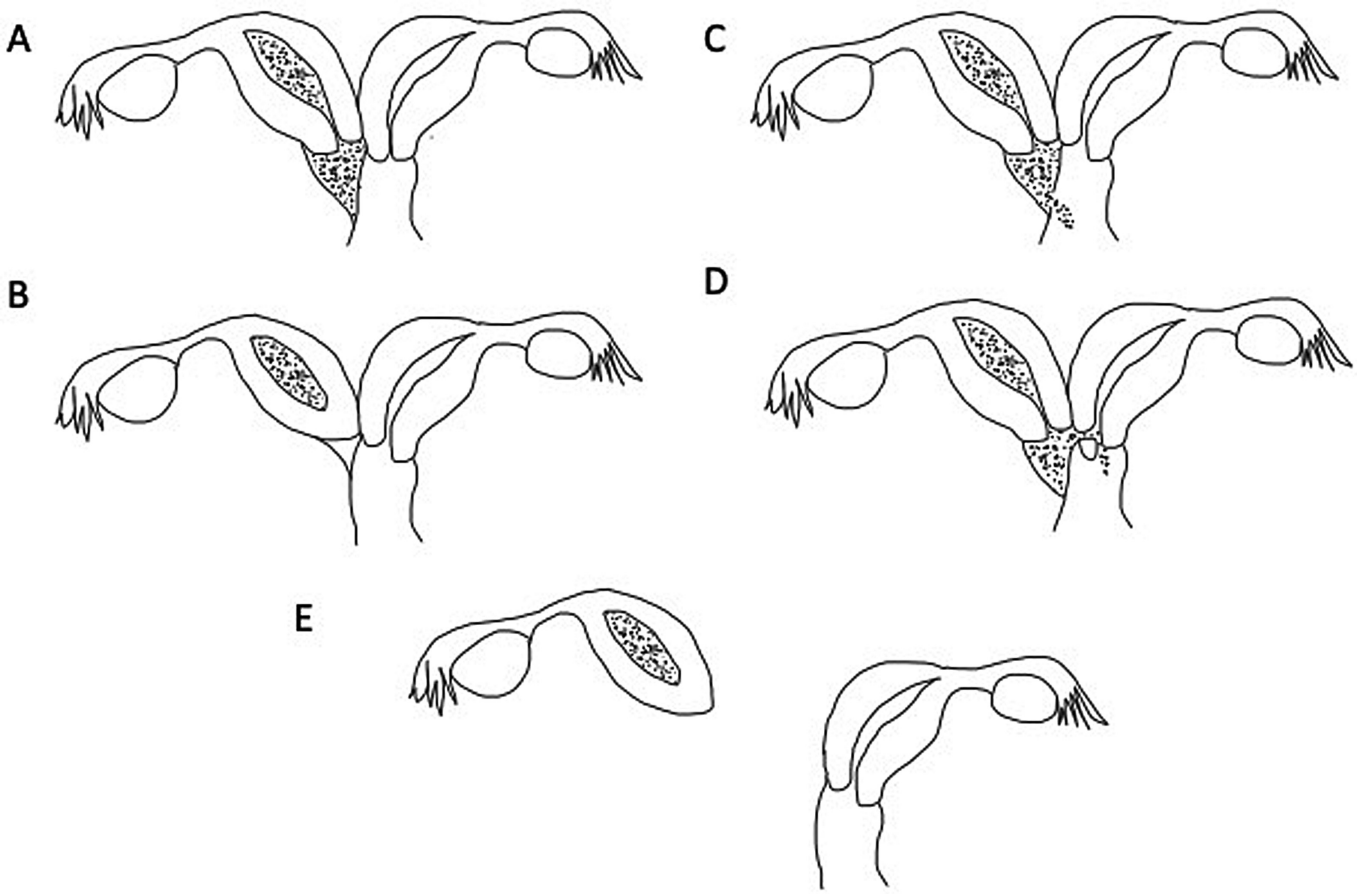
Classification of OHVIRA syndrome based on Zhu et al. 3 ( **A** ) Type 1.1 with complete obstruction of the hemivagina. ( **B** ) Type 1.2, obstructed hemiuterus with cervicovaginal atresia. ( **C** ) Type 2.1 with partial reabsorption of the vaginal septum. ( **D** ) Type 2.2, communicating uteri and incompletely obstructed hemivagina. ( **E** ) Type 1.2 with ectopic obstructed hemiuterus and cervicovaginal atresia, as described in the case report.

The OHVIRA syndrome—obstructed hemivagina and ipsilateral renal anomaly—formerly known as Herlyn–Werner–Wunderlich syndrome, is a rare congenital anomaly of the female genitourinary tract. It is characterized by an obstructed hemivagina and ipsilateral renal anomalies. 1 The underlying mechanism involves a lateral displacement of a paramesonephric/Müllerian duct at approximately 6 weeks' postfertilization, which subsequently disrupts the fusion with the contralateral Müllerian duct and the urogenital sinus. 2 The resulting anatomical structure is characterized by a completely obstructed hemivagina (type 1) or an incompletely obstructed hemivagina (type 2). Type 1 is further subdivided into a variant with blind hemivagina (type 1.1) or cervicovaginal atresia without communicating uteri (type 1.2), whereas type 2 presents with either partial reabsorption of the vaginal septum (type 2.1) or communicating uteri (type 2.2) ( Fig. 1 ). The most common variants are type 1.1 and type 2.1 in about 85% of the cases, with type 1.2 being the rarest variant accounting for less than 5% of the cases. 3 Endometriosis is a potential complication.

OHVIRA syndrome is associated with renal abnormalities in over 98% of patients, with ipsilateral renal agenesis being the most common (93%). Multicystic dysplastic kidney, contralateral duplex kidney, or ectopic kidney are comparatively rare. 4 This is because the Mullerian ducts originate from the same intermediate mesoderm as the mesonephros. 4

Symptoms vary according on the type of obstruction. Girls with complete obstruction (type 1) typically experience pelvic pain due to hematometra shortly after menarche. Women with partial obstruction are usually diagnosed in adulthood because of unspecific symptoms, such as dysmenorrhea. On ultrasound, a cystic pelvic mass may be misdiagnosed as an appendiceal abscess or ovarian cyst. 5 It is important for the attending physician to be aware of this condition. The combination of ultrasound or magnetic resonance imaging (MRI) showing uterus didelphys and hematometra with ipsilateral renal agenesis or other renal abnormalities should allow rapid diagnosis. The general therapeutic principle is to resect the vaginal septum and drain the hematocolpos.

In females, inguinal hernias occur as a result of incomplete closure of the canal of Nuck. The hernia usually involves the ovary and, less commonly, the Fallopian tube or even the uterus. Inguinal hernias are extremely rare in women of reproductive age. 6

## Case Presentation

**Fig. 2:**
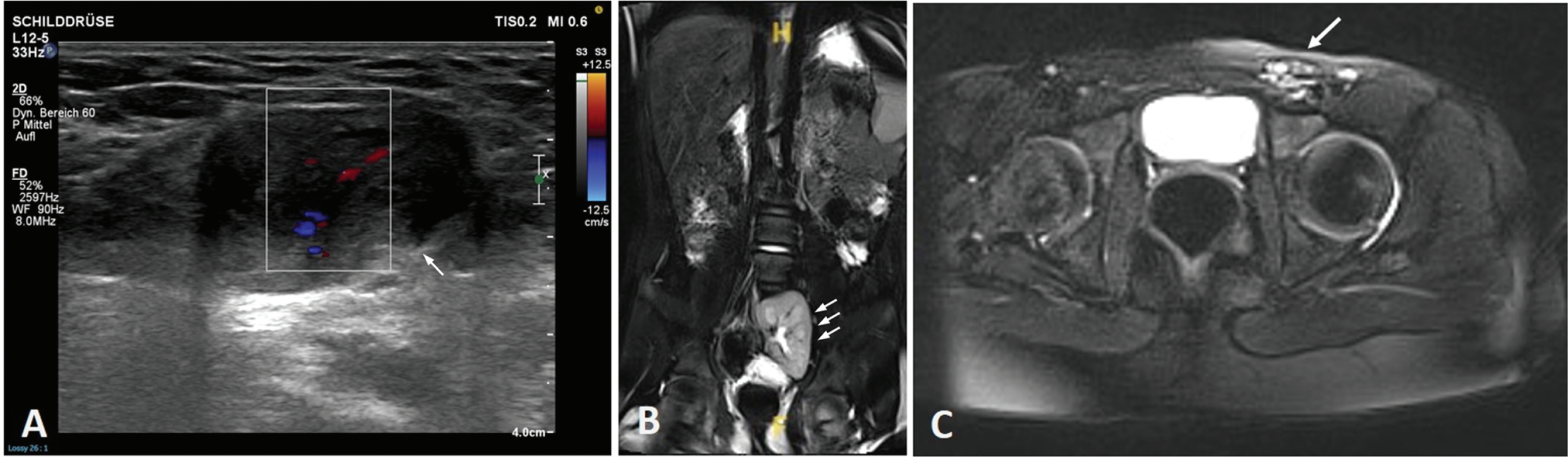
( **A** ) Ultrasound of inguinal ovary in the 12-year-old patient; color Doppler, arrow shows the ectopic ovary. ( **B** , **C** ) Abdominal magnetic resonance imaging of the 8-year-old patient with left ectopic pelvic kidney ( **B** ), three arrows, and inguinal ectopic ovary ( **C** ), arrow.

**Fig. 3:**
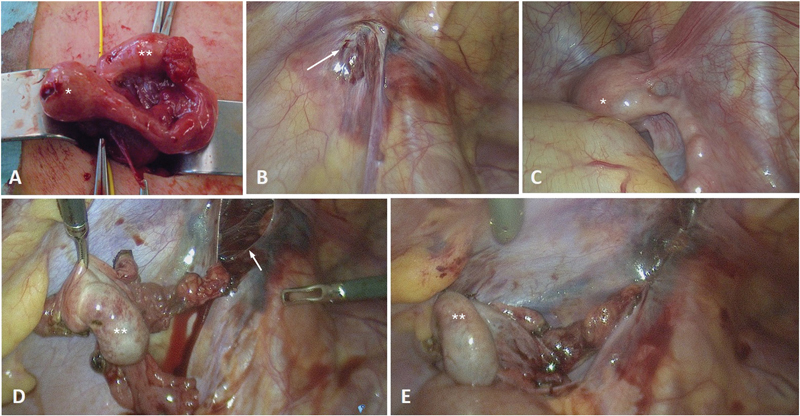
( **A** ) Intraoperative view of the left inguinal atretic hemiuterus (*) and ovary (**). ( **B** ). Closed left internal inguinal ring from laparoscopic view (arrow). ( **C** ). Right-sided orthotopic hemiuterus (*). ( **D** ). Pulled-through left ovary (*) before. ( **E** ) After fixation to the abdominal wall and reclosure of the internal inguinal ring.

A 12-year-old girl presented to our pediatric surgical service with acute groin swelling. An ultrasound showed that the left ovary was located in the left inguinal canal, and color Doppler ultrasound showed normal perfusion ( Fig. 2 ). No further attention was given at this time to the known secondary finding of an ectopic pelvic kidney. An open Inguinal hernia repair was planned as a day case. During the operation, a cyst of Nuck was discovered when the left inguinal canal was opened. The cyst contained an ovary, Fallopian tube, and a hemiuterus, which were not connected to the pelvis or vagina ( Fig. 3 ). A digital vaginal examination revealed an unremarkable vagina and cervix. For further investigation, a diagnostic laparoscopy was performed that revealed a normal right hemiuterus and an unremarkable right ovary. The left inguinal hemiuterus was then resected. To move the left ovary into the pelvis, we transected the closed inner inguinal ring laparoscopically and pulled the ovary through. To prevent torsion, we fixed the ovary to the lateral abdominal wall and ligated the internal inguinal ring with absorbable sutures. The postoperative course was uneventful. The histopathological findings confirmed the presence of a hemiuterus without evidence of a cervix.

The patient's detailed history revealed a history of recurrent groin and pelvic pain since menarche 6 months previously, synchronized with menstrual periods. A previous gynecological examination for these complaints had been inconclusive.

A review of the patient's medical history revealed that an MRI of the abdomen had been performed when the patient was 8 years old to rule out a suspected anomaly of the left ectopic pelvic kidney. In retrospect, the ovary could have been seen in the left groin ( Fig. 1 ).

At follow-up 1 year after surgery, the patient reported pain-free menstruation. On ultrasound, the right ovary was attached to the lateral abdominal wall and color Doppler ultrasound showed normal perfusion.

## Discussion and Conclusion

The OHVIRA syndrome is a rare condition with an estimated incidence of 0.1 to 3.8% in the female population. 7 The classification distinguishes between partial and complete obstruction. 3 In 1,673 reported cases of OHVIRA syndrome, most patients (46.7%) had a completely obstructed hemivagina and a completely isolated hemiuterus (type 1.1), followed by females with a small communication between the hemivaginas (39.2%, type 2.1). According to this meta-analysis, 11.9% of patients had a small communication between the hemiuteri (type 2.2), whereas only 2.2% had cervicovaginal atresia without communicating uteri (type 1.2). 8 In this reported case, the patient presented with a rare condition of type 1.2 condition with simultaneous localization of the ovary, Fallopian tube, and hemiuterus in the canal of Nuck ( Fig. 1E ). There was no history of a sliding hernia in early childhood, and laparoscopy suggested that the herniation must have been present since early infancy as the internal inguinal ring was closed. The MRI performed at the age of 8 years confirmed the preexisting childhood hernia. There was no record or retrospective report of groin pain prior to menarche.

OHVIRA syndrome arises from a failure of embryonic development of the paired Müllerian ducts, which develop on the mesonephric kidneys, and their derivatives, including the Fallopian tubes, uterus, cervix, and the proximal third of the vagina. The maldevelopment is initiated around the sixth week of gestation due to the absence of a physical guide for the Müllerian ducts to descend and fuse with one another and with the urogenital sinus. It is hypothesized that the Wolffian ducts, which are located medially to the Müllerian ducts, act as a physical guide before their regression in embryos with XX chromosomes. Consequently, the Müllerian duct shifts to the ipsilateral side, resulting in a uterus didelphis. Furthermore, the failure of fusion with the urogenital sinus of one Müllerian duct leads to an obstructed hemivagina. 2 Moreover, the formation of the broad ligament is interfered by the missing fusion of the Müllerian ducts leading to a looser suspension of the affected hemiuterus. Dislocation of the ovaries into the inguinal canal may occur in cases of inguinal hernias with missing obliteration of the processus vaginalis. Congenital inguinal ectopy of the ovaries has been described in the literature, particularly in women with Mayer–Rokitansky–Küster–Hauser syndrome and Müllerian anomalies. 9 The round ligaments, which are remnants of the gubernaculum, normally prevent the ovaries from descending inguinally and serve as a ligamentous support for the uterus and ovaries. The gubernaculum arises from the gonadal ridge as undifferentiated mesenchymal tissue and is attached to the gonadal tissue. During the early stages of urogenital development, the gubernaculum differentiates into a connective tissue band that runs from the ovary to the uterus, continuing further through the canal of Nuck to the labia majora. 10 In the absence of gubernaculum-mediated descent inhibition, the ovaries may descend through the inguinal canal. In female mice, the overexpression of insulin-like 3 peptide ( *Insl3* ) has been observed to result in a comparable inguinal descent of the ovaries. 11

In the majority of cases of OHVIRA syndrome, the complexity of surgery is manageable once the diagnosis has been made. If feasible, surgery should be performed as soon as possible after menarche in order to reduce the risk of concurrent endometriosis. Resection of the septum is facilitated by a large distended hematocolpos, so the operation should be performed around the time of menstruation. In patients with OHVIRA type 1.1, 2.1, or 2.2, the vaginal septum is resected via a transvaginal approach; in cases of type 2.2, the uterine septum can also be resected via a minimally invasive laparoscopic approach. 12

For patients with type 1.2, it is recommended to resect the atretic hemiuterus given the difficulty of correcting cervical agenesis surgically. This can be achieved through a laparoscopic or inguinal approach, as demonstrated in the reported case of inguinal ectopic hemiuterus. However, it should be noted that pregnancy outcomes in women with OHVIRA syndrome are underreported in literature. A recent study reported higher incidences of spontaneous abortion, preterm birth, breech presentation, and cesarean delivery compared with the general population. 13

## Notes

New Insights and the Importance for the Pediatric Surgeon The case presented illustrates the diagnostic difficulty of OHVIRA syndrome in general. Due to the combination with an ectopic ovary and obstructed hemiuterus, there is also a risk of confusion with an uncomplicated inguinal hernia with herniated ovary. Even if the findings appear to be clear, a precise medical history and retrospective evaluation of old findings may point the way.
